# From Paris

**Published:** 1892-10

**Authors:** S. Latimer Phillips

**Affiliations:** Paris


### *CORRESPONDENCE.*

---

### FROM PARIS.

PARIS, August 18, 1892.

*Editors Atlanta Medical and Surgical Journal:*

DEAR SIRS—At this season, in Paris as in most large cities, those who can get away have gone to escape the very hot weather, and among them many of the most prominent physicians and surgeons, who well deserve the holiday they take. Upon my arrival in Paris with my friend, Dr. Francis Valk, of New York, the author of a very good work on refraction, I started out to visit the chief eye clinics. We were fortunate in finding most of the men we wished to see, though Dr. Javal, the inventor of that most valuable instrument, the ophthalmometer, for measuring corneal astigmatism, was out of town, so we did not see the instrument in use by him. It is used here, as in America, by many of the leading oculists with much satisfaction. I cannot say anything more here of it, but I hope to give to you before very long a full description of it for the JOURNAL.

Unlike most of the large American cities, we do not find here the large, new, well equipped eye hospitals supported by the city or State, but nearly all are private clinics kept up by the surgeons who organize them. Every man when he obtains a certain amount of celebrity, opens a clinic and keeps it going at his own expense. He begins by renting some building, usually situated in a thickly populated quarter of the city, where the poor come in large numbers to get treatment and medicine free.

Dr. Panas, Professor of Ophthalmology in the Hotel Dieu, one of the largest and best equipped hospitals in Paris, has a large and interesting clinic. I had the pleasure of meeting him, and an interesting conversation upon eye matters with him, though I did not see him operate, as he was taking his holiday for the summer and did not begin his operations before October. His clinic was being run by his two chiefs of clinic. He is a man of fine manner, and speaks English with ease and fluency. Most of the prominent medical men here speak English fairly well, and most of them also speak German. Professor Panas holds his clinics daily, at 9 A. M., and does many operations at which the students and any physician who may so desire are permitted. He is a great advocate of antiseptics in eye surgery, and I believe was the first to use injections of bichloride of mercury in the anterior chamber to wash out the débris, as a part of cataract extraction.

Dr. Galezowski holds his clinics daily at 2 P. M. at 41 Rue Dauphine. Dr. Galezowski possesses a fertile and inventive brain, and is all the time inventing new operations and methods of treatment. He has quite a large clinic, and plenty of material to work upon.

Here one sees large numbers of patients coming in, each with his own box, containing a 2 per cent. solution of arg. nit., a solution of common salt and his camel's hair brushes. These are cases of conjunctival troubles in various stages. The lids are everted, the silver solution brushed over the exposed conjunctiva, and immediately the solution of sodium chloride over that to prevent any undue cauterization. The patients with troubles of the lachrymal sac also have their own probes, which they bring with them daily to be introduced. A method of treatment which I noticed very much in request in this clinic was steaming of the eyes in severe inflammation. In a room provided for this one sees large numbers of people sitting about receiving the vapor bath upon their eyes. This instrument is so arranged that four patients at one time can sit about it and get the bath. A piece of cloth is laid over the eyes, the patient being placed in front of the boiler, a small forehead-rest keeping them at a proper distance, and this steam of vapor is thrown upon each eye. This seems to be very soothing, and a nice method of applying moist heat. I also saw him perform his method of sclerotomy for chronic glaucoma. He prefers this to iridectomy in these cases. The operation consists of four punctures made with a lance-shaped needle knife through the sclera into the anterior chamber above and below, and to the outer and inner side. With the escape of the aqueous of course the tension is lowered. He claims that if necessary the operation can be repeated.

Galezowski, with nearly all of the French surgeons, practices cataract extraction without iridectomy. He now performs sphincterotomy in his extractions, and opens the capsule with the knife before finishing the corneal flap. He claims very great results with his operations. Out of 1,805 extractions in the last ten years, 240 with iridectomy, he only had fifteen cases of hernia of iris, and these mostly due to imprudence of the patients after operation. (De La Sphincterotomie, etc., from Recue d'Ophthalmologie, May, 1892.)

Dr. Galezowski speaks English rather poorly, but he is always ready to give any information that may be desired, and is most pleasant and gracious with all.

Perhaps Dr. DeWecker is generally the best known operator upon the eye in Paris. He has a large clinic in Rue Cherce Midi. I saw him do a number of operations, and had a very pleasant talk with him. He speaks English quite well, and is quite deserving of his great reputation. His clinic is held in the afternoon at four o'clock, but from two to that hour he holds private consultation at Rue Cherce Midi. He is excessively myopic, so much so that in operating he is forced to use but one eye, and so nearly approaches his head to that of the patient that it is difficult at times to see what he doing. He left Paris before I did to join Grand Duke Carl, who is an intimate friend of his, and who is himself quite an accomplished oculist. Dr. DeWecker informed me that he would spend the month of September in Bayreuth, where he would do many operations. He thought perhaps he would do over three hundred. He makes his annual trip there at this season.

I will not be able to write more now, but will try to write to you again from Berlin and London. Very truly yours,

S. LATIMER PHILLIPS.

